# Supplementary figures and images for: The impact of nutritional status in nivolumab-treated patients with advanced esophageal cancer
Source: PLoS One. 2023 May 5;18(5):e0285365. doi: 10.1371/journal.pone.0285365 (PMC10162549; doi:10.1371/journal.pone.0285365)

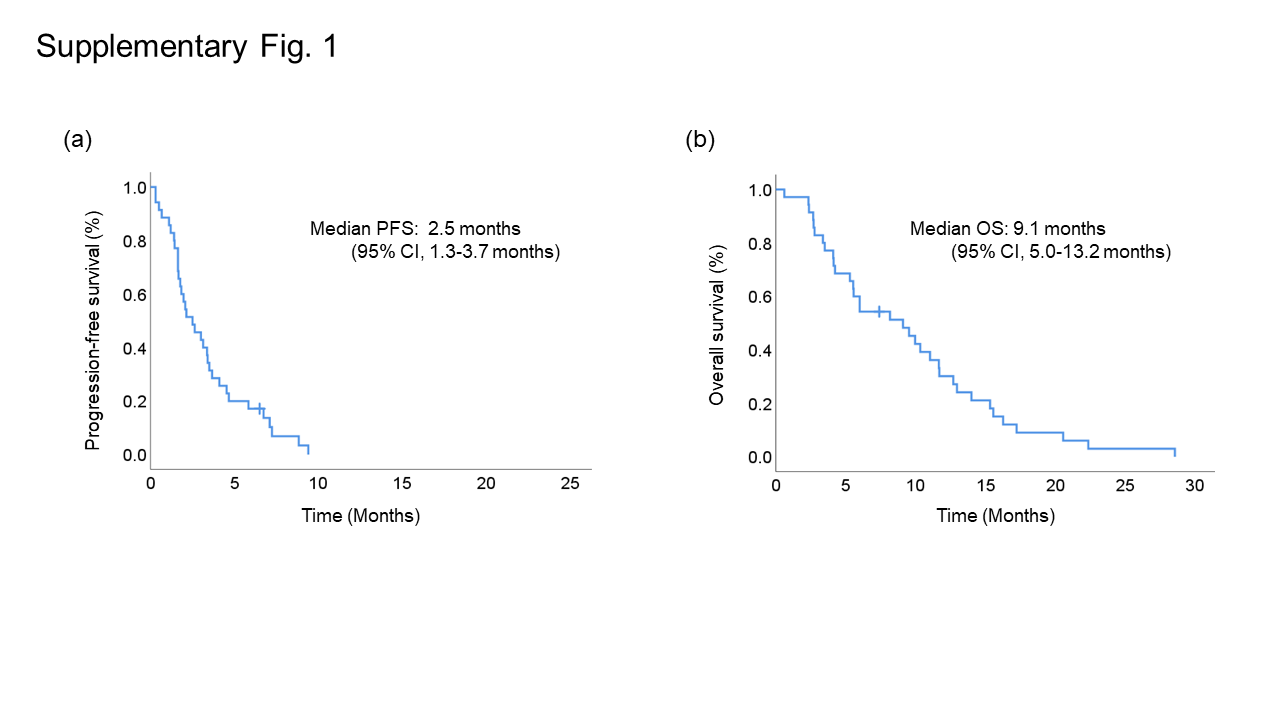

Supplement: S1 Fig — Kaplan–Meier analysis of (a) PFS and (b) OS. (TIF) [file pone.0285365.s001.TIF]

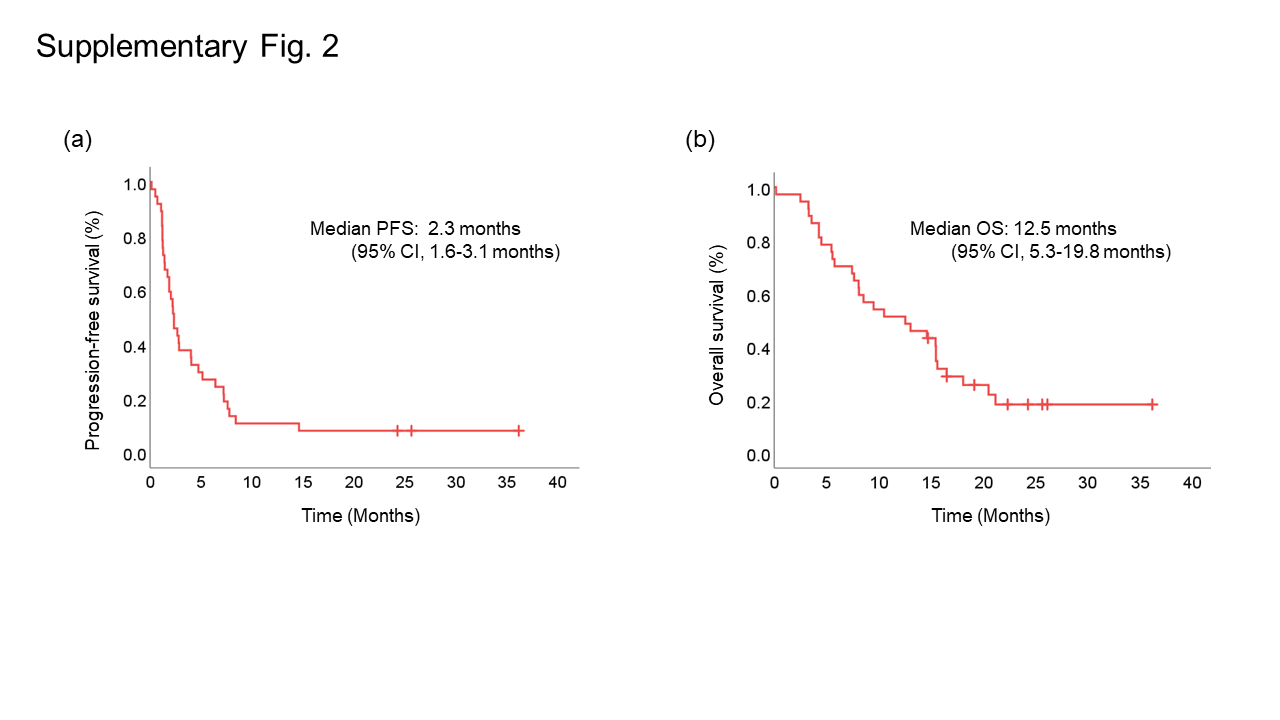

Supplement: S2 Fig — Kaplan–Meier analysis of (a) PFS and (b) OS. (TIF) [file pone.0285365.s002.TIF]

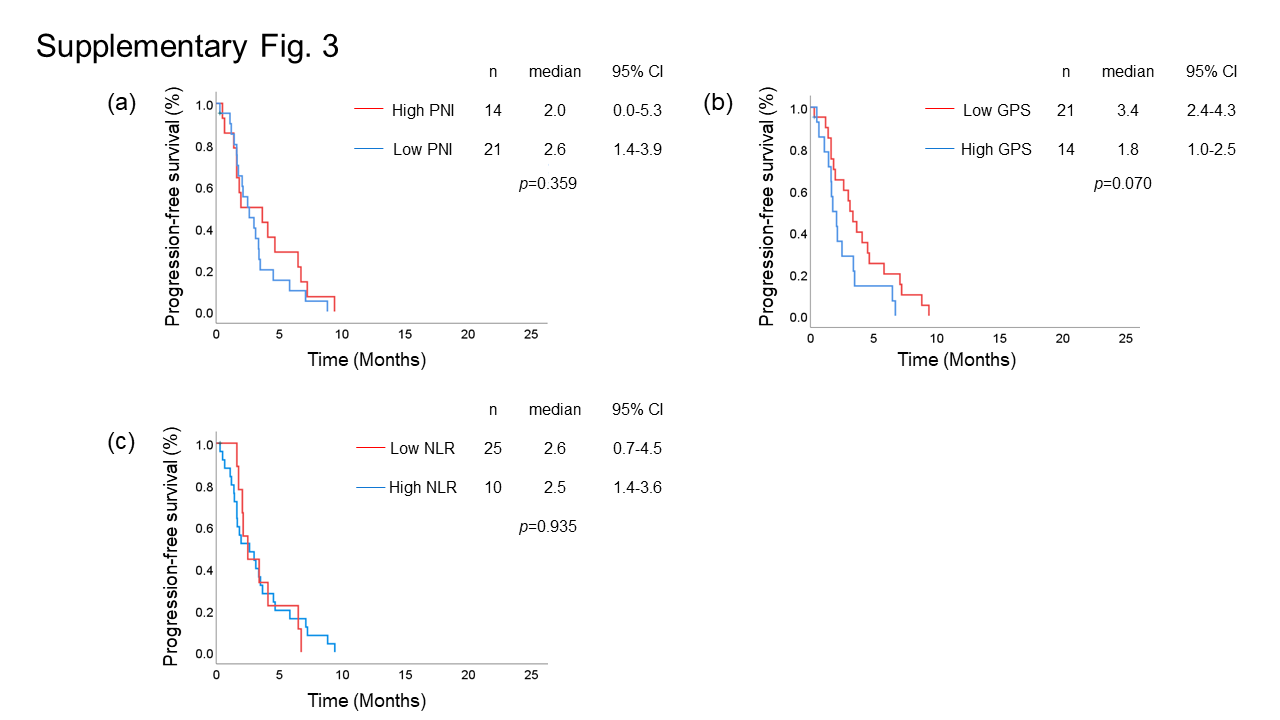

Supplement: S3 Fig — Kaplan–Meier analysis of PFS in the taxane cohort: (a) high PNI (red line) and low PNI (blue line), (b) low GPS (red line) and high GPS (blue line), and (c) low NLR (red line) and high NLR (blue line). (TIF) [file pone.0285365.s003.TIF]

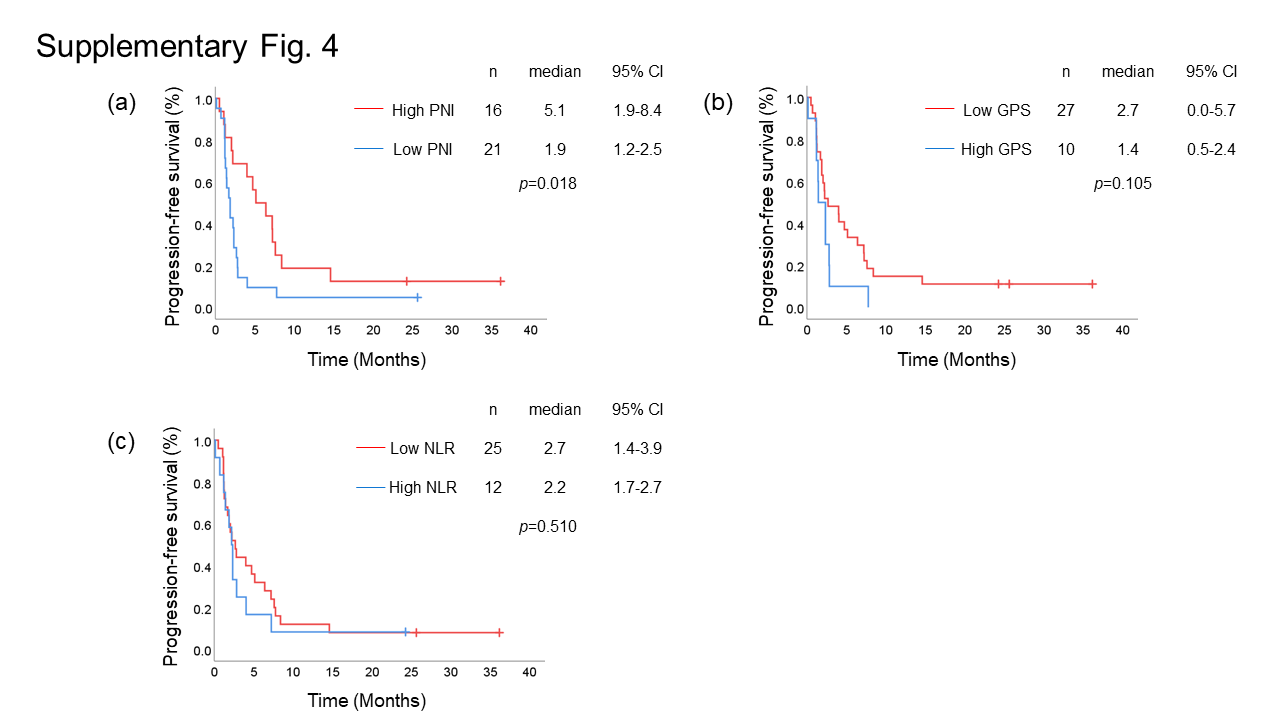

Supplement: S4 Fig — Kaplan–Meier analysis of PFS in the nivolumab cohort: (a) high PNI (red line) and low PNI (blue line), (b) low GPS (red line) and high GPS (blue line), and (c) low NLR (red line) and high NLR (blue line). (TIF) [file pone.0285365.s004.TIF]

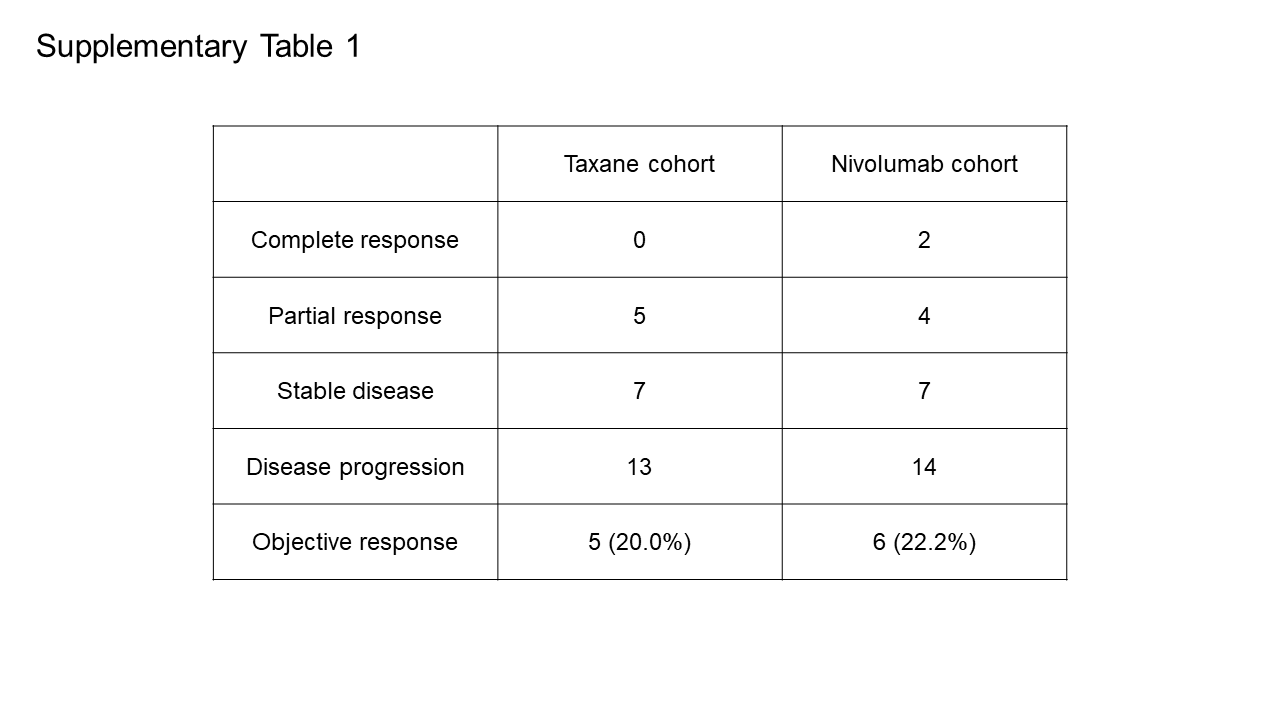

Supplement: S1 Table — (TIF) [file pone.0285365.s005.TIF]
