# Supplementary material for: The impact of nutritional status in nivolumab-treated patients with advanced esophageal cancer
Source: PLoS One. 2023 May 5;18(5):e0285365. doi: 10.1371/journal.pone.0285365 (PMC10162549; doi:10.1371/journal.pone.0285365)
Supplement: Attachment — Submitted filename: minnor comment.docx [file pone.0285365.s006.docx]

Thank you for your valuable consideration.

Although the number of cases is small, it is interesting to compare the data between taxane and nivolumab regarding the relationship between nutritional status and therapeutic effect.

Comment 1.

There is a similar study with a large number of patients on nivolumab, so please cite and consider the content.( Inflammatory prognostic factors in advanced or recurrent esophageal

squamous cell carcinoma treated with nivolumab, Cancer Immunology, Immunotherapy

<https://doi.org/10.1007/s00262-022-03265-7>)

Comment 2.

Enter the number of patients after each cohort in the first row of table.1.
